# Hemorrhage or thrombosis? Adrenal involvements in a patient with antiphospholipid syndrome

**DOI:** 10.1515/rir-2025-0032

**Published:** 2025-12-27

**Authors:** Lingshan Liu, Qin Wang, Ying Jiang, Can Huang

**Affiliations:** Department of Rheumatology and Clinical Immunology, Peking Union Medical College Hospital (PUMCH), Chinese Academy of Medical Sciences & Peking Union Medical College, Beijing 100730, China; Department of Radiology, Peking Union Medical College Hospital (PUMCH), Chinese Academy of Medical Sciences & Peking Union Medical College, Beijing 100730, China; State Key Laboratory of Complex Severe and Rare Diseases, Beijing 100730, China; National Clinical Research Center for Dermatologic and Immunologic Diseases (NCRC-DID), Ministry of Science & Technology, Beijing 100730, China; Key Laboratory of Rheumatology and Clinical Immunology, Ministry of Education, Beijing 100730, China

**Figure 1:**
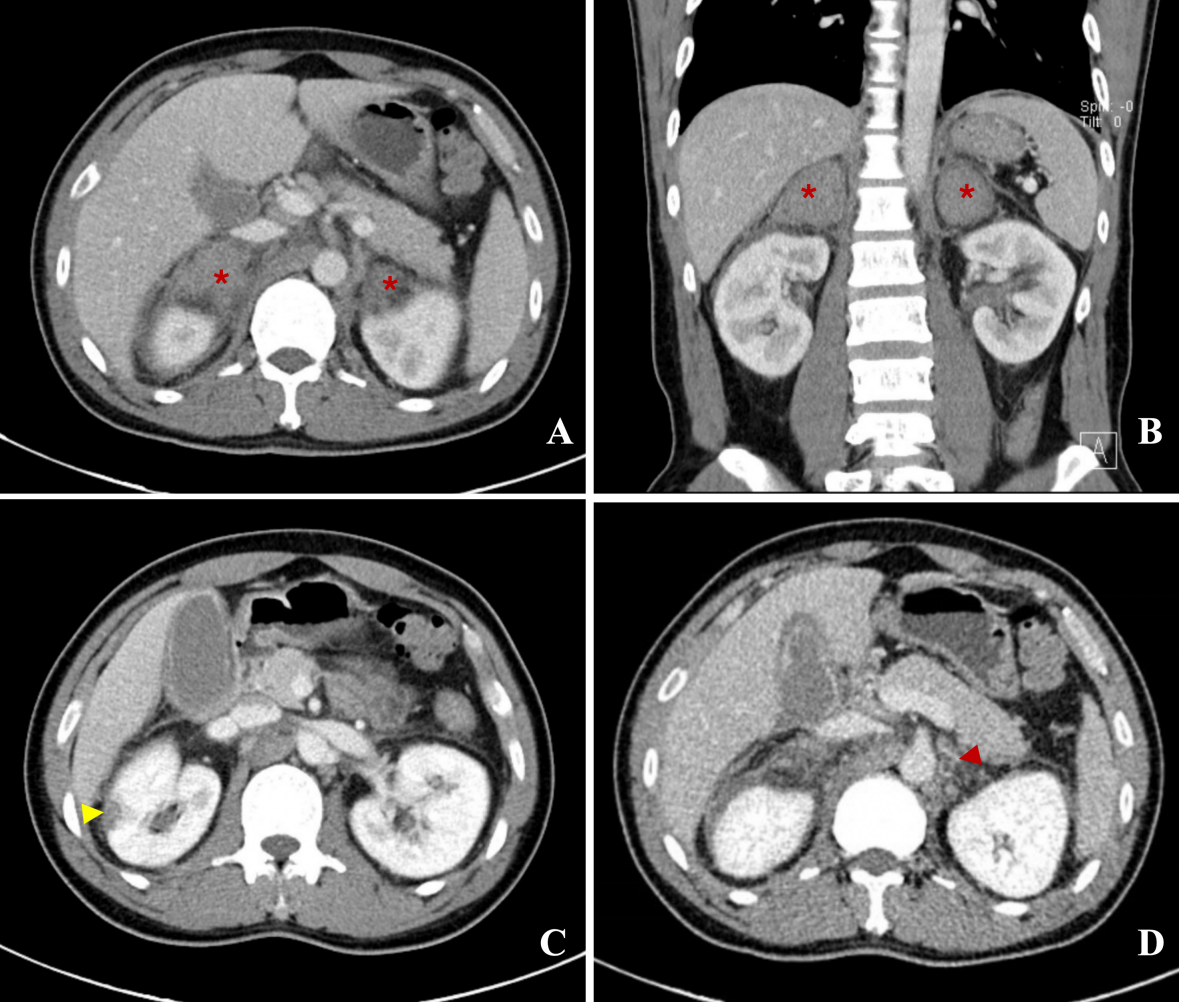
A, B: Transverse and coronal section of enhanced abdominal tomography (CT), showed clearly bilateral adrenal hemorrhage (AH), manifesting as slightly high-density shadow with no enhancement, marked with asterisk. C: A wedge-shaped hypo-enhanced lesion indicating ischemic change was seen at marginal of right kidney, marked with yellow arrow. D: A patchy low-density shadow was found in left adrenal vein, marked by red arrow, which might be a thrombosis.

A 39-year-old man was admitted with persistent abdominal and back pain, accompanied by nausea and vomiting for 19 days. Five years prior, he had been diagnosed with popliteal vein thrombosis and was subsequently confirmed to have antiphospholipid syndrome (APS). He had been on long-term oral warfarin therapy, which he had discontinued without medical guidance. At the onset of current symptoms, laboratory investigations at a local hospital revealed hyponatremia (serum sodium 123 mmol/L), markedly prolonged activated partial thromboplastin time (APTT, 102.3 s), and elevated inflammatory markers (C-reactive protein [CRP], 47.55 mg/L), and mild enlargement of the left adrenal gland on contrast-enhanced abdominal computed tomography. Low molecular weight heparin (LMWH) was interrupted due to evidence of adrenal hemorrhage (AH) and hematuria. Upon admission to our hospital, repeat enhanced CT revealed new-onset right-sided AH in addition to the left, appearing as a slightly hyperdense, non-enhancing area (Figure 1A, 1B, asterisk). Another wedge-shaped hypo-enhanced lesion was seen at the right kidney margin, suggesting ischemia (Figure 1C, yellow arrow). On delayed imaging, a patchy low-density shadow in the left adrenal vein (Figure 1D, red arrow) raised suspicion of a thrombosis. The diagnosis of APS was confirmed by persistently high titers of anticardiolipin antibody-IgG (ACL-IgG, 135.1 IU/mL), anti-β2 glycoprotein I antibody-IgG (aβ2GPI-IgG, 825.9 IU/mL), and lupus anticoagulant (LA, 2.16). Serum cortisol was decreased and adrenocorticotropin (ACTH) was increased. Microvascular involvement was also detected in myocardium (elevated enzymes and ischemic changes on perfusion magnetic resonance imaging [MRI]), skin (painful purplish-red rash on toes), and pancreas (elevated pancreatic enzymes), leading to the diagnosis of catastrophic APS (CAPS). Macrovascular involvement was thoroughly excluded. Despite the presence of bilateral AH, therapeutic LMWH was resumed, and methylprednisolone pulse therapy (1000 mg/d for 3 days) was applied, followed by oral prednisone (1.0 mg/kg/d, gradually tapered). Symptoms improved and laboratory parameters returned to normal. LMWH was transitioned to warfarin (international normalized ratio [INR] maintained 2–3), and cyclophosphamide (CTX, 100 mg/d orally) and hydroxychloroquine (HCQ, 0.2 g twice a day) were added. The patient’s condition remained stable during 4-month follow-up.

AH is a rare clinical manifestation of APS (~1%),^[1]^ yet holds diagnostic importance. It has been incorporated as a microvascular manifestation in the 2023 revised APS classification criteria ^[2]^ Although APS is a thrombotic disease, adrenal involvement in APS often presents as hemorrhage, attributed to the glands’ unique vascular architecture. Adrenal arteries form a rich supply, while venous drainage is limited, predisposing to thrombosis-induced congestion and hemorrhage.^[3]^ Thrombosis in adrenal veins is difficult to visualize due to their small caliber, making early-stage imaging findings obscure, when combined with nonspecific symptoms, complicating the early detection of AH and adrenal vein thrombosis. However, AH as a manifestation of microvascular APS (MAPS), often implies an underlying CAPS, and is linked to elevated mortality.^[4]^ Therefore, AH signs warrant immediate APS screening. Prompt anticoagulantion is critical, and glucocorticoid pulse therapy and immunosuppressants such as cyclophosphamide might also be necessary if CAPS is suspected.^[5,6]^ This case underscores the clinical significance of APS-related AH and highlights the importance of its early recognition and timely intervention.
